# Experimental Research on the Preparation of K_2_CO_3_/Expanded Vermiculite Composite Energy Storage Material

**DOI:** 10.3390/ma15103702

**Published:** 2022-05-22

**Authors:** Dequan Zou, Xiangji Yue, Tianyi He, Jianan Ding, Dechun Ba

**Affiliations:** School of Mechanical Engineering and Automation, Northeastern University, Shenyang 110819, China; zoudequan1220@163.com (D.Z.); 1970264@stu.neu.edu.cn (T.H.); jnding@yeah.net (J.D.); dchba@mail.neu.edu.cn (D.B.)

**Keywords:** thermochemical energy storage, salt hydrates, vacuum impregnation, K_2_CO_3_, expanded vermiculite

## Abstract

Thermochemical adsorption energy storage is a potential energy utilization technology. Among these technologies, the composite energy storage material prepared by K_2_CO_3_ and expanded vermiculite (EVM) shows excellent performance. In this paper, the influence of the preparation process using the impregnation method and vacuum impregnation method on K_2_CO_3_/EVM composite material is studied. The preparation plan is further optimized with the solution concentration and the expanded vermiculite particle size as variables. In the experiment, mercury intrusion porosimetry (MIP) is used to measure the porosity and other parameters. Additionally, with the help of scanning electron microscopy (SEM), the morphological characteristics of the materials are obtained from a microscopic point of view. The effects of different preparation parameters are evaluated by comparing the experimental results. The results show that the K_2_CO_3_ specific gravity of the composite material increases with the increase of the vacuum degree, up to 70.440 wt.% (the vacuum degree is 6.7 kPa). Expanded vermiculite with a large particle size (3~6 mm) can carry more K_2_CO_3_, and content per cubic centimeter of K_2_CO_3_ can be as high as 0.466 g.

## 1. Introduction

The Intergovernmental Panel on Climate Change (IPCC) Special Report on Global Warming of 1.5 °C pointed out that reducing the average temperature rise from 2.0 °C to less than 1.5 °C will greatly reduce the risks of sea-level rise and urban high temperature [1]. Solar energy, as a renewable and clean energy source, used in building heating can help control global temperature rise [2]. However, the solar radiation is intermittent, and it has insufficient thermal energy supply in winter [3]. Therefore, the salt hydrates seasonal energy storage technology has become a hot spot in the field of energy utilization. The study found that the salt hydrate K_2_CO_3_ has good reversibility, a low price and non-corrosiveness. Combining K_2_CO_3_ and porous matrix expanded vermiculite (EVM) into a composite material can highlight the advantages of K_2_CO_3_ [4,5].

For the K_2_CO_3_ reaction equilibrium formula, see Formula (1). During the dehydration reaction, K_2_CO_3_·1.5H_2_O loses water and absorbs heat. During the hydration reaction, K_2_CO_3_ combines with the water vapor medium to form a hydrate and release heat. The temperature required for K_2_CO_3_ dehydration and hydration reaction is relatively low, which is suitable for medium- and low-temperature heat storage systems such as building heating [6].

K_2_CO_3_·1.5H_2_O(s) = K_2_CO_3_(s) + 1.5H_2_O(g) (1)

There is a wide range of methods for preparing composites, among which the impregnation method is widely used. Yu et al. [7] immersed two kinds of silica gels with different average pore diameters in LiCl solution and separated them with a vacuum filter to obtain LiCl/silica gel composite materials. They found that silica gels with larger pore sizes can carry more inorganic salts. Courbon et al. [8] prepared silica gel and LiBr composite adsorbent based on the multi-step incipient wetness method. Posern et al. [9] put Attapulgite granulate with a porosity of 74.3% in the mixed solution of MgSO_4_ andMgCl_2_, and they found that the exothermic heat of the obtained composites increased with increasing MgCl_2_ content. Shere et al. [10] used the porous structure of zeolite to prepare mixed inorganic salt composites by impregnation. The experiment found that the energy storage density is maximum when the proportion of MgCl_2_ and MgSO_4_ is 50%. Chan et al. [11] immersed the zeolite in 46 wt.% CaCl_2_ solution and filtered it after 24 h to obtain a CaCl_2_/zeolite composite material, in which the Ca content reached 41.5 mol%. Grekova et al. [12] used the impregnation method to immerse the EVM which had been dried for 16 h in the LiCl solution and then dried again at 160 °C to obtain a LiCl/EVM composite with a salt content of 59 wt.%. In order to prevent particle agglomeration and improve mass transfer, Veselovskaya et al. [13] soaked the EVM matrix in the BaCl_2_ aqueous solution to disperse the BaCl_2_ particles in the pores. Zhang et al. [14] immersed the dried EVM in SrBr_2_ solutions with different mass concentrations for 48 h. After testing multiple composite materials, they found that the SrBr_2_/EVM composite material with a salt content of 63.02 wt.% is the best. With the development of the vacuum industry, the combination of vacuum technology and impregnation method forms a vacuum impregnation method, which provides a different impregnation environment for composite materials. Li et al. [15] prepared paraffin/EVM composites by vacuum impregnation and found that EVM can be loaded with up to 67% paraffin. Xu et al. [16] immersed paraffin in the lamellar gap of EVM based on a vacuum environment and learned that this composite material has excellent thermal stability according to the thermogravimetric analysis (TGA). Karaipekli et al. [17] also selected the vacuum impregnation method when immersing the eutectic mixture of fatty acids into the EVM, and measured that the maximum mass fraction of the impregnant can reach 40%.

Based on the preparation process of the impregnation method and the vacuum impregnation method, this paper uses EVM as the matrix to prepare K_2_CO_3_/EVM composite material. The influence of the operation process and the degree of vacuum on the K_2_CO_3_/EVM composite material during dipping is studied, and the feasibility of the preparation schemes are further discussed with the solution concentration and EVM particle size as variables. In addition, the distribution of K_2_CO_3_ in the composite material is also described. The research results can provide a reference for the preparation of similar types of composite materials.

## 2. Materials and Instruments

**Figure 1:**
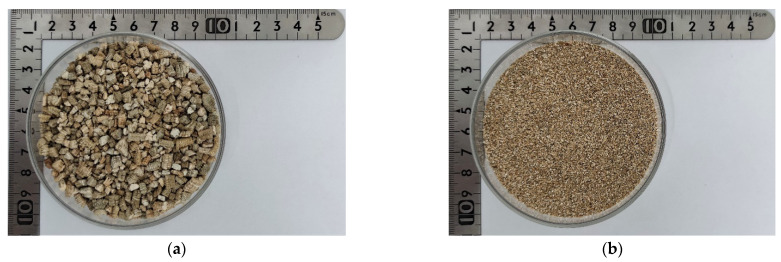
The appearance of the EVM. (**a**) E1 (3~6 mm); (**b**) E2 (0.4~0.8 mm).

In order to study the effects of operating procedures and differences in experimental variables, expanded vermiculite and anhydrous K_2_CO_3_ are used to prepare K_2_CO_3_/EVM composite materials. Expanded vermiculite (EVM), which is obtained from vermiculite ore [(Mg,Fe,Al)_8_[(Al,Si)_4_O_10_](OH)_2_·4H_2_O] by thermal expansion method or chemical expansion method [18,19], has the characteristics of high porosity, low density, fire resistance, and is widely used in chemical, construction, metallurgy, environmental protection [20,21,22,23]. In this paper, two types of EVM are selected: E1 (3~6 mm) and E2 (0.4~0.8 mm). The appearance of EVM is shown in Figure 1. Anhydrous K_2_CO_3_ is white granular, which crystallizes into white translucent crystals after hydration reaction. In this experiment, analytically pure anhydrous K_2_CO_3_ is used.

Mercury intrusion porosimetry (MIP, AutoPore IV 9500) is used to measure the EVM of two particle sizes to obtain EVM porosity, pore size and other parameters. Take about 0.05 g of the EVM sample dried at a high temperature into the dilatometer, seal and weigh it. The experimental pressure range is 3.58~2.27 × 10^5^ kPa, and the data are collected and analyzed.

**Figure 2:**
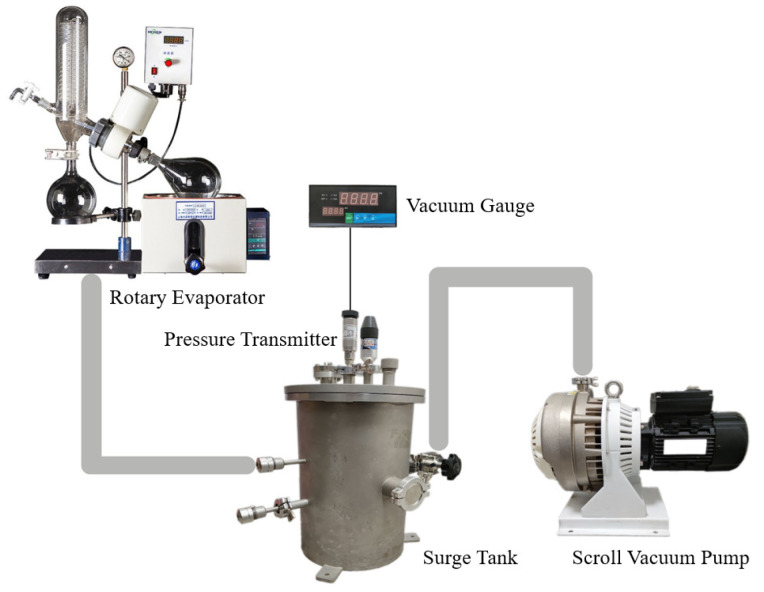
The K_2_CO_3_/EVM composite material preparation equipment.

The K_2_CO_3_/EVM composite material preparation equipment is composed of a rotary evaporator, vacuum gauge, pressure transmitter, scroll vacuum pump and surge tank, as shown in Figure 2. The materials are mixed in the rotating bottle of the rotary evaporator, and the other devices provide different vacuum degrees according to the experimental requirements. In the process of preparing the composite material, an electronic balance is used for weighing, and its division value is 0.001 g.

A scanning electron microscopy (SEM, Hitachi S-4800) is used to observe the surface and cross-sectional microstructure of EVM and composite material to study the distribution of K_2_CO_3_. The sample after high-temperature drying is fixed on the sample stage with electrically conductive adhesive and is conductively treated by sputtering Pt. The sample is sent into the sample chamber and vacuumize to 2 × 10^−3^ Pa, and the SEM image is obtained under an acceleration voltage of 5.0 kV.

## 3. Preparation of Composite Material

**Figure 3:**
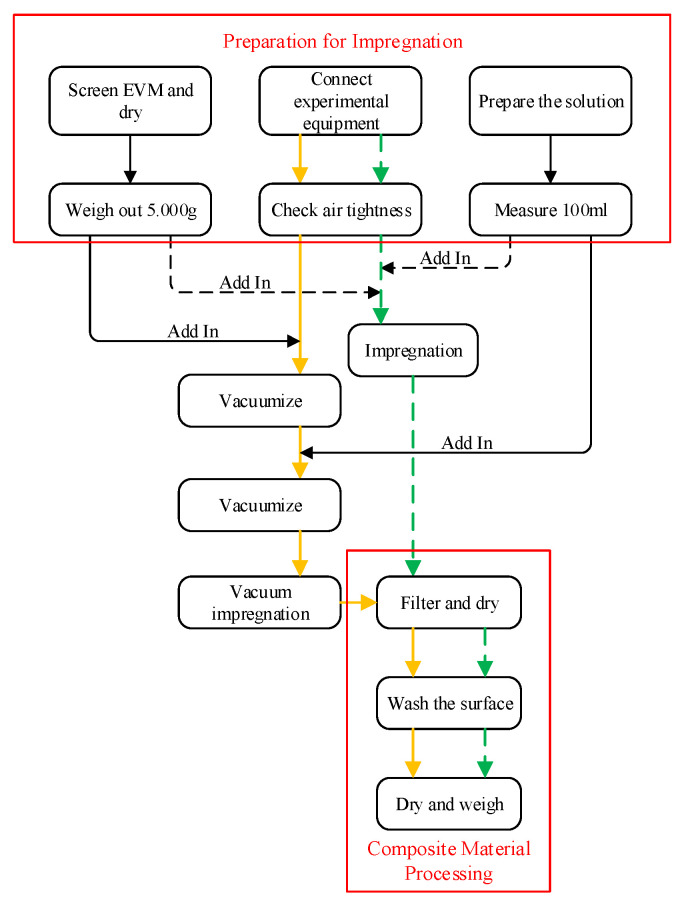
Composite material preparation process (┄: impregnation preparation process; —: vacuum impregnation preparation process).

According to the relevant descriptions of the above-mentioned preparation methods of composite materials, the preparation process of composite materials is designed based on the experimental objectives, as shown in Figure 3. When preparing the composite material based on the impregnation method, the preparations such as drying the EVM, connecting the experimental equipment, and configuring the K_2_CO_3_ solution with anhydrous K_2_CO_3_ and deionized water are first carried out. Then put the K_2_CO_3_ solution and EVM into a rotating bottle to mix and get impregnated. In the impregnation process, some samples added agitation and other operations. After the impregnation, the excess K_2_CO_3_ solution was filtered with a screen and dried for the first time. Then the excess K_2_CO_3_ was cleaned from the surface of the sample with deionized water. Sögütoglu et al. [24] believed that high-temperature heating can remove the KHCO_3_ impurities formed when K_2_CO_3_ is stored in the air. Therefore, the composite material will finally be dried in a high-temperature environment of 120 °C for 8 h to remove moisture and possible KHCO_3_ impurities. When the composite material is dried, it is weighed using an electronic balance. The operation steps of the vacuum impregnation method in the preparation for impregnation and composite material processing stages are the same as those of the impregnation method. However, in the impregnation process, the vacuum impregnation method provides a certain degree of vacuum by twice vacuuming for the EVM to absorb the K_2_CO_3_ solution. The salt content in the sample is expressed by the specific gravity *η* of K_2_CO_3_ in the composite material. The larger the value of *η*, the more K_2_CO_3_ contained in the unit mass sample, and the more conducive to energy storage. The calculation formula of *η* is:

(2) $$\eta =\frac{m_{KE}-m_{E}}{m_{KE}}\times 100\%$$

wherein, *m_KE_* is the mass of the composite material after drying, g; *m_E_* is the mass of the EVM, g.

In this paper, three groups of composite materials are prepared, namely the KE1, KE2 and KE3 series, all of which are prepared with 5.000 g EVM and 100 mL K_2_CO_3_ solution. The KE1 series of samples are used to study the influence of the operation and vacuum degree on the K_2_CO_3_/EVM composite material during impregnation, and to analyze the distribution of K_2_CO_3_ with the help of SEM. The KE2 series samples are prepared by 0.4~0.8 mm EVM, and are compared with the KE1 series samples to study the influence of the particle size of the EVM on the preparation of composite material. The KE3 series samples change the mass concentration of the K_2_CO_3_ solution during the vacuum impregnation process and study the influence of the solution concentration on the K_2_CO_3_ specific gravity and K_2_CO_3_ distribution of the composite material. The main operations and formulas of each group of composite materials are shown in Table 1, where 1.12 g/mL solution is saturated K_2_CO_3_ solution, and 0.125~0.750 g/mL solution is unsaturated K_2_CO_3_ solution. Sample KE1-1 is the no. 1 sample prepared from 3~6 mmEVM and saturated K_2_CO_3_ solution, and other samples are similar.

## 4. Results and Discussion

### 4.1. The Specific Gravity of K_2_CO_3_

#### 4.1.1. Operation

In order to seek an ideal preparation process, in other words, to prepare composite material with high K_2_CO_3_ specific gravity in the shortest time, there are 6 different schemes being designed for comparison. The specific data are shown in Table 2 among which KE1-1~KE1-3 are prepared by the impregnation method, and the KE1-4~KE1-6 samples are prepared by the vacuum impregnation method.

Table 2 shows that the salt mass carried by the KE1-4~KE1-6 samples is more than twice the mass of the EVM, resulting in the specific gravity of K_2_CO_3_ in the composite material as high as 70.440 wt.%, which is about 20 wt.% higher than the three samples prepared by the impregnation method. It can be seen that the vacuum degree is very beneficial to increase the salt content of the composite material. Comparing the samples of KE1-1~KE1-3, it is found that the specific gravity of K_2_CO_3_ does not change significantly, though the impregnation time of KE1-1 is 48 h and KE1-3 has a stirring operation. The *η* values of the three samples prepared by the vacuum impregnation method are all around 70 wt.%, and the increased operation of stirring and evaporating water does not have a great impact on the composite material. It can be seen from the above results that vacuum is the main factor to increase the specific gravity, and the use of vacuum impregnation can obtain composite material with high K_2_CO_3_ specific gravity in a relatively short period of time.

#### 4.1.2. Vacuum Degree

**Figure 4:**
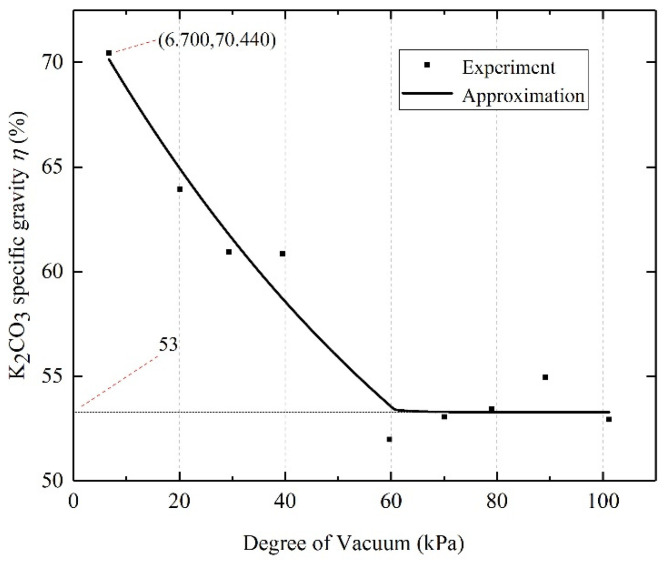
K_2_CO_3_ specific gravity change diagram with vacuum degree.

The vacuum degree is the main influencing factor in the preparation of composite material, so the relationship between its numerical value and the specific gravity of K_2_CO_3_ becomes extremely important. The graph of the specific gravity of K_2_CO_3_ with the vacuum degree is shown in Figure 4. The data of each point in the figure are obtained based on the vacuum impregnation method (KE1-4 sample preparation process). Figure 4 shows that when the vacuum degree is higher than 60 kPa, as the vacuum degree decreases (the preparation pressure increases), the specific gravity of K_2_CO_3_ in the composite material shows a decreasing trend. When the preparation pressure exceeds about 60 kPa, the vacuum environment will not greatly promote the absorption of K_2_CO_3_ solution by the EVM, and the specific gravity of K_2_CO_3_ is stable at about 53 wt.%.

#### 4.1.3. EVM Particle Size

The main operations and data of KE2 series samples are shown in Table 3. For the composite material prepared by 0.4~0.8 mm EVM (E2), even with vacuum impregnation, the specific gravity of K_2_CO_3_ is only 48.347 wt.%, which is lower than the KE1-4 sample (3~6 mm EVM:E1) of the same preparation process. It can be seen that the large-particle-size EVM can carry more K_2_CO_3_. From the microscopic level, the different content of K_2_CO_3_ carried by EVM with different particle sizes should be related to EVM pore parameters. Therefore, the porosity and other parameters of E1 and E2 are measured by mercury intrusion porosimetry, and the MIP test results are shown in Table 4.

**Figure 5:**
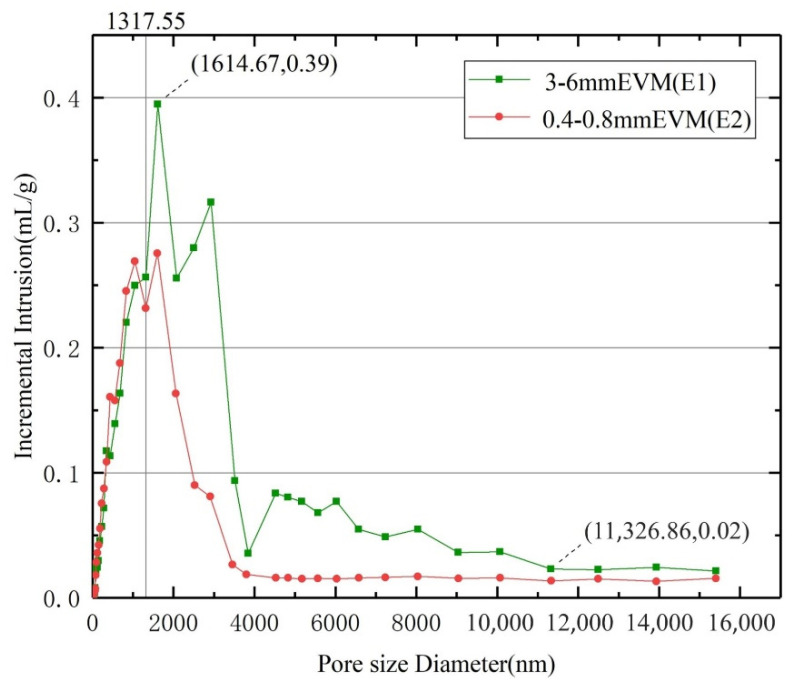
Incremental intrusion vs pore size.

Table 4 shows that both E1 and E2 have a high porosity of more than 95% and a total pore area of about 18.5 m^2^/g. However, the difference in average pore diameter values of them is larger. The average pore diameter of E1 is as high as 1068.24 nm, which is about 23.952% larger than E2. It can be seen that the pore diameter is the main factor that affects how much K_2_CO_3_ the EVM carries. Figure 5 shows that within the pore size range of 1317.55 to 11,326.86 nm, the incremental intrusion of E1 is significantly higher than that of E2. As the data continue to rise, the incremental intrusion by E1 can reach as high as 0.39 mL/g, which is about 43.116% larger than E2. It can be seen that E1 has more pores with a pore diameter larger than 1317.55 nm. Under vacuum, E1 can absorb a large amount of K_2_CO_3_ solution and obtain higher K_2_CO_3_ specific gravity.

**Figure 6:**
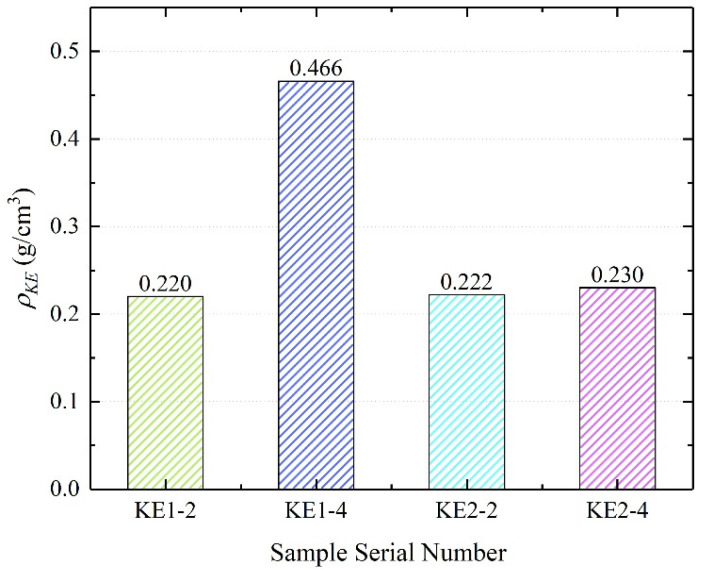
*_KE_* calculation result.

Taking into account the difference in E1 and E2 bulk density (*ρ_B_*, g/cm^3^), the ratio (*ρ_KE_*, g/cm^3^) of K_2_CO_3_ mass to EVM bulk volume is used to compare the salt content of composite materials with different particle sizes:

(3) $$\rho_{KE}=\frac{m_{K_{2}CO_{3}}}{V_{0}}=\frac{m_{KE}-m_{E}}{m_{E}}\times \rho$$

where *V_0_* is the bulk volume of EVM, cm^3^; and *m_K2CO3_* is the mass of K_2_CO_3_ in the composite material, g. It can be known from the calculation results of *ρ_KE_* in Figure 6, the *ρ_KE_* value of the KE2-2 sample prepared by the impregnation method is 0.222 g/cm^3^, which is about 1.18% larger than that of the KE1-2 sample. However, the *ρ_KE_* value of the KE1-4 sample prepared by the vacuum impregnation method can reach 0.466 g/cm^3^, which is much higher than that of other samples. It can be seen that the composites prepared from large-particle-size EVM and K_2_CO_3_ solutions in a vacuum environment can store more inorganic salts. The higher the content of inorganic salt per unit volume, the more energy is absorbed, which is more conducive to improving the practicability of the material.

#### 4.1.4. Solution Concentration

**Figure 7:**
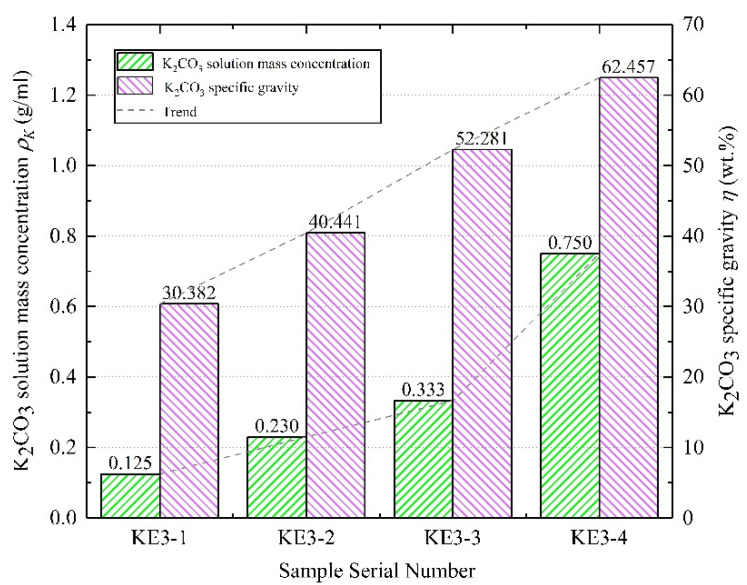
KE3 series samples preparation data.

Four K_2_CO_3_ solutions of different concentrations are used to prepare K_2_CO_3_/EVM composite materials. The specific preparation data are shown in Figure 7. As the mass concentration of K_2_CO_3_ solution increases, the specific gravity of K_2_CO_3_ in the composite material gradually increases. The solution concentration of the KE3-3 sample is 0.103 g/mL higher than that of the previous sample, and the *η* difference between the two samples can reach 11.84 wt.%. The solution concentration of the KE3-4 sample is 0.417 g/mL higher than that of the KE3-3. However, the specific gravity of K_2_CO_3_ only increased by 10 wt.%. It can be seen that the specific gravity of K_2_CO_3_ in the composite material does not increase linearly with the increase of the solution concentration, instead, there is a trend of decreasing growth. This phenomenon is mainly because EVM absorbs K_2_CO_3_ solution through capillary action, but the absorbing capacity of the capillarity will decrease with the increase of the concentration of the solution [25].

### 4.2. Distribution of K_2_CO_3_

#### 4.2.1. Vacuum Degree

**Figure 8:**
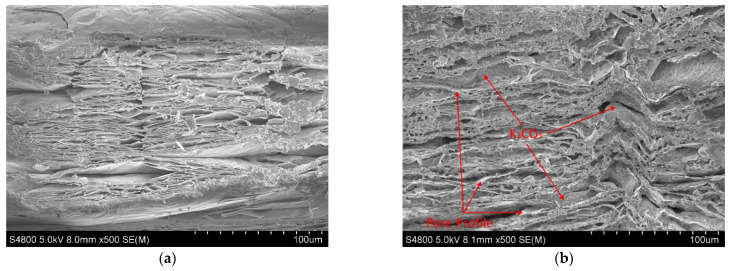

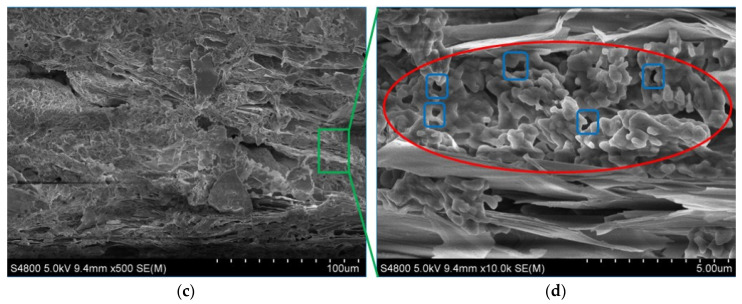
SEM images of EVM, KE1-2 and KE1-4 samples surfaces. (**a**) EVM; (**b**) KE1-2; (**c**) KE1-4; and (**d**) KE1-4 Porosity.

The K_2_CO_3_/EVM composite material prepared by the vacuum impregnation method can carry more K_2_CO_3_. In order to study the distribution of K_2_CO_3_ in the EVM of composite materials prepared in different environments, the EVM is used as a blank control to observe the surface and cross-sectional morphologies of KE1-2 and KE1-4 samples. The SEM images of the surface of EVM, KE1-2 and KE1-4 samples are shown in Figure 8. Figure 8a shows that in EVM without K_2_CO_3_, pores of different sizes are clearly visible on the surface. In contrast, in the KE1-2 sample prepared based on the impregnation method (Figure 8b), most of the space in the pores is occupied by K_2_CO_3_ (dark), and only the outline of the pores (bright white) can be observed on the surface. The surface of the KE1-4 sample prepared by the vacuum impregnation method (Figure 8c) is covered by more K_2_CO_3_, and it is almost impossible to observe the pore profile. Magnifying the pore by 10.0k times, it is found that the pores are filled with a large amount of K_2_CO_3_ (red circles in Figure 8d), and there are small gaps between K_2_CO_3_ particles that can be used for water vapor medium circulation (blue rectangles).

**Figure 9:**
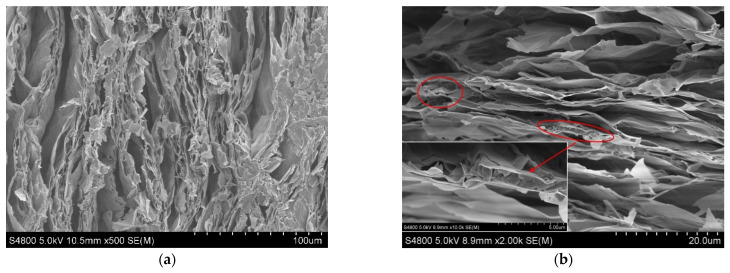

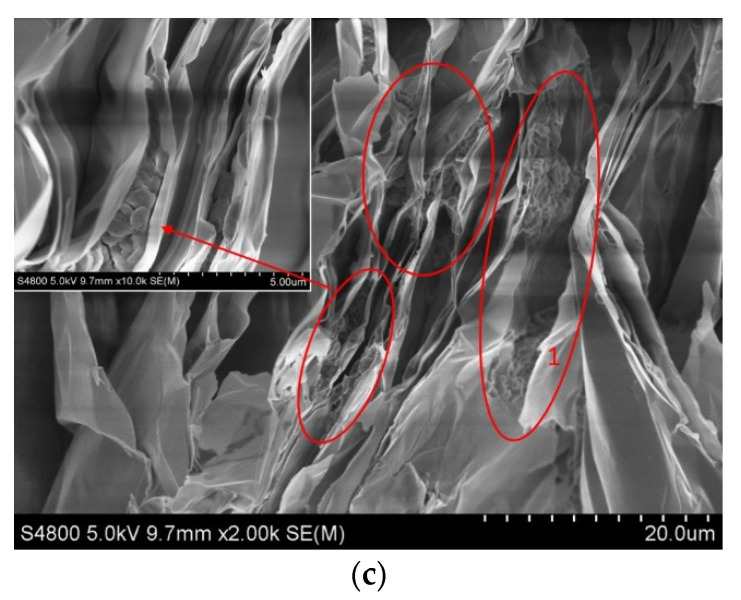
SEM images of EVM, KE1-2 and KE1-4 samples cross-sections. (**a**) EVM; (**b**) KE1-2; and (**c**) KE1-4.

The SEM images of the cross-sections of EVM, KE1-2 and KE1-4 samples are shown in Figure 9. The lamellas in the EVM section are thin and dense (Figure 9a), and there are random connections between the lamellas that form pores of different sizes. Only a small number of pores in the cross-section of the prepared KE1-2 sample contain K_2_CO_3_, while the observed K_2_CO_3_ coverage areas at the section of the KE1-4 sample are more (red circles in Figure 9b,c). Although the KE1-4 sample carries a large amount of K_2_CO_3_, there are also many unfilled pores inside. Among the pores containing K_2_CO_3_ in the KE1-4 sample, most of the K_2_CO_3_ in the large pores adhered to the wall of the pore, and the small pores are basically filled with K_2_CO_3_. The K_2_CO_3_ in the small pores is in the form of particles, and the medium circulation gap between the particles is not large.

#### 4.2.2. Solution Concentration

**Figure 10:**
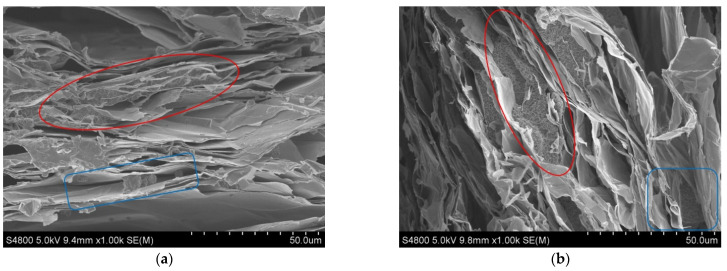

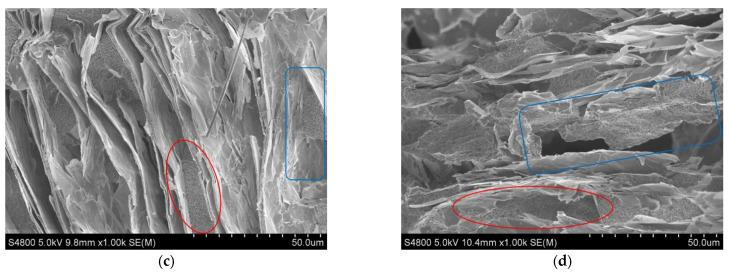
SEM image of KE3 series sample cross-section. (**a**) KE3-1; (**b**) KE3-2; (**c**) KE3-3; and (**d**) KE3-4.

The SEM images of the cross-section of the KE3 series are shown in Figure 10. Figure 10 shows that although the concentration of the solution changes during the preparation of the composite material, the K_2_CO_3_ in the macropores of the KE3 series samples adheres more to the wall of the pores, leaving a larger medium flow area (blue rectangles), and it is similar to the morphology in the macropores of the KE1-4 sample (1 in Figure 9c). The distribution of K_2_CO_3_ in the small pores has also not changed with the different concentrations of the solution, and it is basically filled (red circles). It can be seen that the morphology of K_2_CO_3_ in the pores of the composite material will not change significantly with the decrease in the solution concentration. Using a high-concentration solution to prepare composite materials can make the EVM carry more K_2_CO_3_ and improve the practicability of the material.

## 5. Conclusions

K_2_CO_3_/EVM composite material, as one of many substances in thermochemical adsorption energy storage technology, has outstanding advantages such as low price, non-corrosiveness and toxicity. Its dehydration temperature is lower than 120 °C, which is very suitable for use with solar thermal utilization technology. In order to obtain a more practical composite material, based on the impregnation method and the vacuum impregnation method, the influence of the preparation process, the particle size of the expanded vermiculite and the solution concentration on the K_2_CO_3_/EVM composite material are studied, and the conclusions are as follows:

The vacuum degree is the main factor in order to increase the specific gravity of K_2_CO_3_. When the vacuum degree is lower than 60 kPa, the specific gravity of K_2_CO_3_ in the composite material is stable at about 53 wt.%, and there is no significant change. When the vacuum degree is higher than 60 kPa, the specific gravity of K_2_CO_3_ in the composite material shows an upward trend with the increase of the vacuum degree, up to 70.440 wt.% (the vacuum degree is 6.7 kPa).

Large-particle-size EVM can carry more K_2_CO_3_. As 3~6 mm EVM (E1) has more large pores with pore diameters of 1317.55~11,326.86 nm, E1 can absorb more K_2_CO_3_ solution than E2 (0.4~0.8 mm EVM). Among the samples, the K_2_CO_3_ content per cubic centimeter of the KE1-4 sample prepared by E1 can be as high as 0.466 g.

Part of the pores in the composite material does not contain K_2_CO_3_ particles. Among the pores containing K_2_CO_3_, the K_2_CO_3_ in the large pores is more adhered to the wall of the pore, while the small pores are basically filled with K_2_CO_3_. Under the effect of vacuum, more K_2_CO_3_ is distributed on the surface and inside of the EVM, as a result, the composite material has a higher specific gravity of K_2_CO_3_. The increase in the concentration of the impregnating solution promotes the increase of the specific gravity of K_2_CO_3_ in the composite material, but the distribution of K_2_CO_3_ particles in the pores does not change.

## Figures and Tables

**Table 1:** The main operation and formula of composite materials.

Series	Sample No.	Main Operation	Operation during Impregnation	EVM Particle Size D/mm	Mass Concentration of K_2_CO_3_ Solution *ρ_k_*/(g/mL)
KE1	KE1-1	Impregnation for 48 h	─	3~6	1.12
	KE1-2	Impregnation for 6 h	─		
	KE1-3	Impregnation for 6 h	Mix		
	KE1-4	Vacuum impregnation for 6 h	─		
	KE1-5	Vacuum impregnation for 6 h	Mix		
	KE1-6	Vacuum impregnation for 6 h	Mix + evaporated water		
KE2	KE2-2	Impregnation for 6h	─	0.4~0.8	1.12
	KE2-4	Vacuum impregnation for 6 h	─		
KE3	KE3-1~4	Vacuum impregnation for 6 h	─	3~6	0.125~0.750

**Table 2:** KE1 series sample data.

Sample No.	KE1-1	KE1-2	KE1-3	KE1-4	KE1-5	KE1-6
Vacuum degree (absolute pressure) *P_abs_*/kPa	101.2	101.2	101.2	6.7	10.0	8.5
Quality after drying *m_KE_*/g	10.081	10.621	10.483	16.915	15.532	16.799
K_2_CO_3_ accounts for the specific gravity of composite material *η*/wt.%	50.402	52.923	52.304	70.440	67.808	70.236

**Table 3:** KE2 series of major operations and sample data.

Sample No.	Vacuum Degree (Absolute Pressure) *P_abs_*/kPa	Quality after Drying *m_KE_*/g	K_2_CO_3_ Accounts for the Specific Gravity of Composite Material *η*/wt.%
KE2-2	101.2	9.522	47.490
KE2-4	7.8	9.680	48.347

**Table 4:** MIP test results.

Sample No.	Porosity/%	Average Pore Diameter D_A_/nm	$\mathrm{Total}\;\mathrm{Pore}\;\mathrm{Area}\;S/(m^{2}/g)$	Bulk Density *ρ_B_*/(g/cm^3^)
E1	96.2082	1068.24	18.418	0.1956
E2	98.4322	861.82	18.568	0.2460

## Data Availability

The data presented in this study are available on request from the corresponding author.
